# Electroretinography and suicidal behaviors: a systematic review

**DOI:** 10.1186/s12888-023-05453-w

**Published:** 2023-12-15

**Authors:** Mark Mohan Kaggwa, Sebastien Prat, Arianna Davids, Amara Robbins, Bailea Erb, Mini Mamak, Gary Andrew Chaimowitz, Andrew T. Olagunju

**Affiliations:** 1 Department of Psychiatry and Behavioural Neurosciences, McMaster University, Hamilton, ON Canada; 2 Forensic Psychiatry Program, St. Joseph’s Healthcare, Hamilton, ON Canada; 3 Department of Psychiatry, Mbarara University of Science and Technology, Mbarara, Uganda; 4 Discipline of Psychiatry, University of Adelaide, Adelaide, SA 5000 Australia

**Keywords:** Electroretinography, Suicidal behaviors, Neurotransmitters and Review

## Abstract

**Background:**

Electroretinogram (ERG) is one of the tools used to investigate the electrophysiological underpinnings of mental health illnesses and major clinical phenomena (e.g., suicide) to improve their diagnosis and care. While multiple studies have reported specific ERG changes among individuals with suicidal behaviors, we know of no review that has been done to characterize their findings to inform future research.

**Methods:**

This review included available literature concerning ERG and suicidal behaviors. The paper’s first section briefly overviews the theoretical basis of ERG and neurotransmitters involved in suicidal behaviors. The second section describes the findings of a review of studies reporting ERG findings among individuals with suicidal behaviors.

**Results:**

Most reviewed studies reported normal amplitude and implicit time of the a-waves, but the latency in individuals with suicidal behaviors was lower than normal. Additionally, the b-waves amplitude was reduced, but the implicit time and latency were increased. The b-a amplitude ratio and oscillatory potential were decreased.

**Conclusion:**

Despite identifying certain ERG correlates with suicidal behaviors in the existing studies, there is a need for adequately powered and methodologically robust studies to advance clinical translation.

**Supplementary Information:**

The online version contains supplementary material available at 10.1186/s12888-023-05453-w.

## Introduction

Suicide is a public health issue of concern, with over 700,000 individuals dying by suicide yearly, but proportionally more people demonstrating suicidal behaviors [1, 2]. The etiological underpinnings and ramifications of suicide are often construed with the biopsychosocial theory, and various models such as the stress diathesis model have been used to explain the etiology and risks for suicide and suicidal behaviors [3–5]. However, a suicide attempt is the strongest predictor of suicide [2], and approximately 44% of people with suicidal ideations contact their healthcare providers within the month before a suicide attempt [2]. While clinical and other forms of assessments have been proposed for early identification of suicidal risk, many assessments for suicidal behaviors are subjective and characterized by high rates of false negatives or non-disclosure, especially to clinicians [6]. For this reason, several objective methods (including genomic, proteomics, clinical, electrophysiological, and laboratory tests, etc.) are explored to assess their potential benefits for screening, diagnostic evaluation, and prediction of suicidal behaviors [7–10].

There are many electrophysiological techniques that have the potential to detect suicidal behaviours. In particular, several studies have explored techniques related to testing brain electrical activities using electroencephalography (EEG) including quantitative EEG, polysomnographic examination and quantitative sleep EEG, auditory evoked potential loudness, and other event-related potentials) to detect suicide [10]. The findings obtained with these techniques are sometimes coupled with radiological testing [9], which support the idea that executive function directly impacts emotion regulation in individuals with suicidal behavior. When emotion regulation is disrupted, individuals with suicidal behavior turn to maladaptive procedures that reduce their ability to cope with emotional stress. Emerging evidence has also shown important correlates of suicidal behavior using electroretinography (ERG) —involves the measurement of the electrical response of the eye’s light-sensitive cells, called rods and cones. Notably, the ERG has shown a reduction of the b-wave latency among individuals with suicidal behaviors [7].

Despite the potential benefits of using these non-invasive electrophysiological methods to detect suicidal behaviors, introducing these methods into routine clinical practice has been problematic, given their economic costs and dependence on the operators’ skills. For example, EEG is limited to measuring electrical potentials from the area of cortical gray matter and hippocampi, while electrical potentials from subcortical structures, such as the basal ganglia and brainstem nuclei, cannot be measured by EEG because their electrical potentials do not reach the surface of the head [11]. Comparatively, majority of the available electrophysiological research and reviews have explored the role of EEG and radiological testing in suicidology, but few have focused on ERG correlates of suicidal behaviors. Hence, this review focuses on the ERG correlates of suicidal behaviors. The review is divided in two sections, first we provided a brief overview of the theoretical basis of ERG and neurotransmitters involved in suicidal behaviors, and the second section described results from a review of available studies about suicidal behaviors and ERG.

### Overview of the theoretical basis of ERG and neurotransmitters involved in suicidal behaviors

#### Electroretinography

**Fig. 1:**
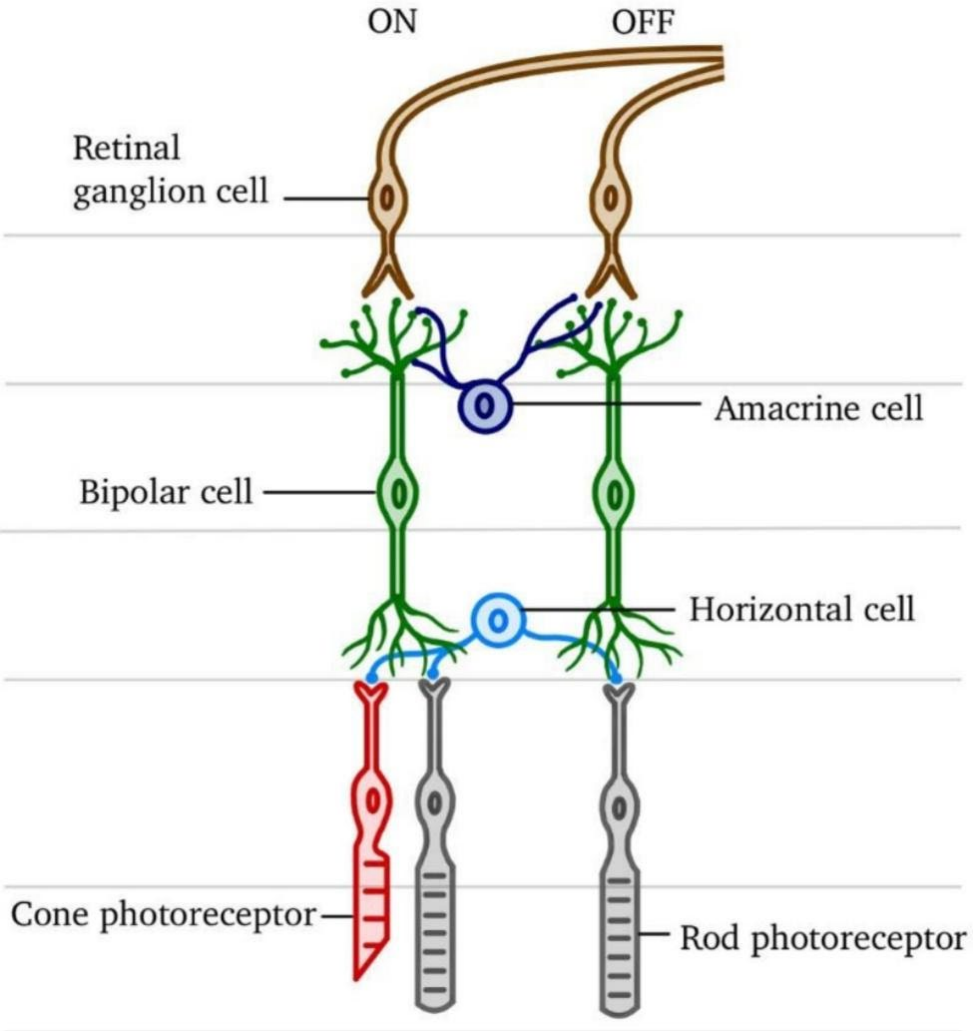
Retina cells. Representation of the retinal network and its different pathways for visual information processing. Visual information is processed by the ‘columnar unit,’ which includes photoreceptors, bipolar cells, and retinal ganglion cells. Horizontal and amacrine cells modulate the synaptic signaling between photoreceptors and bipolar cells and between bipolar cells and retinal ganglion cells. Retinal ganglion cell axons initially form bundles of unmyelinated fibres at the retinal nerve fibre layer (RNFL), which will then exit the eye and form the myelinated optic nerve. Rod and cone photoreceptors mediate two distinct pathways of light responses: scotopic and photopic vision. In particular, the cone pathway can be additionally distinguished according to ON and OFF responses or waves, which are interconnected and differentially modulated by glutamate neurotransmission

**Fig. 2:**
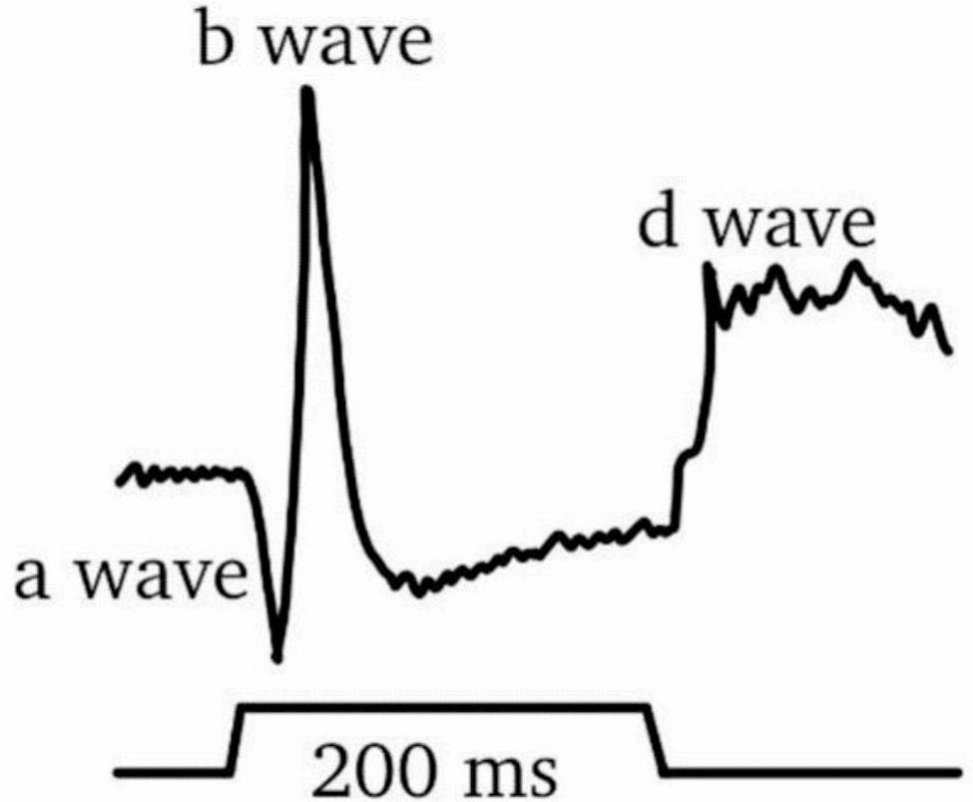
The classical ERG graphs showing the different waves.

The ERG is a specialized electrophysiologic tool that records the electrical responses of retinal cell types, including photoreceptors (rod and cones), bipolar and amacrine cells, and ganglion cells [12, 13] (Fig. 1). ERG is particularly useful for determining the generalized retinal function amongst individuals. ERG detects the retinal responses through the placement of a few small electrodes around the eye area and a succession of brief flashes of light. Common parameters measured by ERG include the amplitude and latency of a and b- waves, while less common parameters include a photopic negative response (PhNR) and oscillatory potentials (OP) (Table 1). These parameters will be described below in further detail (Fig. 2).

The International Society for Clinical Electrophysiology of Vision (ISCEV) is a global association that standardises clinical protocols for electrophysiological examination. The first ISCEV guidelines came out in1989 [14, 15], and have been regularly updated. The latest update from 2022 [16] outlines six standardized full-field ERG protocols for isolating the electrical activity of the rods and cones. The protocols are named based on the eye’s adaptation state (whether dark-adapted or light-adapted) and the stimulus strength (light intensity). Four protocols are dark-adapted, and two are light-adapted [12]. An extended photopic on-off protocol for ERG has been used to assess retina function in studies analyzing psychiatric conditions [9]. The specifications of the protocol are as follows: (1) the stimulus duration is between 150-200ms, (2) the stimulus should be white or chromatic, with the same-coloured background, (3) the strength of the stimulus must be between 150 and 350 cd m^− 2^ with the background luminance at 30 cd m^− 2^, and (4) the rate of stimulus presentation should be less than or equal to two per second, with an inter-stimulus interval of greater than or equal to five seconds [17]. The protocol emphasises the on-and-off response to light and produces various waves based on the retina cells’ response. The waves include a, b, and d waves. Both a and b waves are on-responses while d-wave constitute the off-response. The a-wave (the initial negative deflection) corresponds to the early hyperpolarization of the rod and cone photoreceptors. The b-wave is the positive deflection following the a-wave. B-waves originate from the depolarization of inner retinal glia and bipolar cells. However, off-bipolar and horizontal cells may influence their amplitude and shape [17]. The off-response or d-wave is a positive polarity component in response to stimulus offset [17]. The d-wave initial rapid phase originates from off-bipolar cell activity, but cone photoreceptors contribute to the later slow phase, and on-bipolar cells act in an opposite polarity direction. See Table 1.

**Table 1:** ERG wave forms

Wave	Functional retina cells represented	Neuronal activity	Possible neurotransmitters
a-wave	Photoreceptors cells layer	Hyperpolarization of photoreceptors → initial negative deflection	Dopamine (rod sensitivity) [13] Glutamate [18]
b-wave	Inner nucleus layer cells (muller and bipolar cells)	Depolarization of inner retinal Müller and bipolar cells → positive deflection following the a-wave	Dopamine (D1R to D5R) Serotonin (Tph1) [13]
d-wave	Off-bipolar cells, cone photoreceptors, on-bipolar cells	Off-bipolar cell activity → initial rapid phase Cone photoreceptor → later slow phase On-bipolar cells → act in an opposite polarity direction	Dopamine (D1R to D5R) [13] Imbalances between GABA and glutamate [19]
Oscillatory potentials	Amacrine cells, some vascular cells		Glutamate (VGLUT3) [18] GABA (many different varieties of Amacrine cells) [18]

Other aspects of the waves include Oscillatory potentials (OPs) and PhNR. OPs are visible at greater signal intensities and represent high-frequency rhythmic wavelets on the rising slope of the b-wave. OPs reflect the electrical activity of inner retinal feedback synaptic circuits, namely amacrine cells and some vascular functions. PhNR is the negative deflection that follows the b-wave and represents light-adaption. The PhNR originates from the retinal ganglion cells in response to a brief flash.

The waves are analyzed based on amplitude, implicit time, latency, and b-wave to a-wave ratio [20]. The amplitude is the maximal light-induced voltage (electrical response) generated by the different retinal cells. The a-wave amplitude measurement starts from the baseline of the ERG to the negative trough of the a-wave, and the b-wave amplitude starts from the trough of the a-wave to the subsequent b-wave peak. Like the a-wave, the PhNR amplitude measurement starts at the baseline of the ERG to the negative trough of the PhNR. Implicit time, or time-to-peak, is the time needed for the electrical response to reach maximum amplitude. Implicit time is measured from the stimulus onset to the peak of the corresponding wave component. Thus, the implicit time reflects the rate of electrical signal conduction. Unlike implicit time, latency is the time from stimulus onset to response onset. The b-wave to a-wave ratio provides an index of inner to outer retinal function. However, the accuracy of the findings of the ERG is affected by various factors [20].

#### Factors affect the findings of an ERG

In their work, Asanad and Karanjiah [20] have previously outlined factors that interfere with ERG findings. They are outlined as follows: (i) Use of non-standardized testing conditions, such as sub-optimal lighting, recording environment, pupil size, flash intensity, and altering duration of light or dark adaptation; (ii) Uncorrected refractive errors; (iii) Ocular media opacification; (iv) Electrode-based artifacts due to poor contact with skin or cornea, unstable position, high electrical impedance, and incorrect placement; (v) Eye movement like blinking during ERG recording; (vi) Reduced electrical response because of aging; (vii) Response depression with anesthesia; (viii) Diurnal fluctuation; and (ix) Variability in recordings between different device types.

#### Neurotransmitters within the retina responsible for the ERG findings

Based on human and animal studies, various neurotransmitters have been identified in the retina, mainly in the amacrine cells [18]. The following are neurotransmitters in the Amacrine cells: GABA, Glycine, Acetylcholine, Dopamine, Serotonin, Substance P, VIP, Somatostatins, and Nitric oxide. However, the dominant neurotransmitter is Glutamate [18, 21]. The oscillatory potentials reflect the activities of the amacrine cells [20]. The bipolar cells have receptor channels for glutamate, but their axonal endings have receptors and channels for GABA (A, B, and C types), dopamine (D1), and glycine. The ganglion cells have diverse receptors as bipolar cells with additional acetylcholine receptors. Polymorphism in genes is responsible for the neurotransmitters, their receptors, and channels identified in the retina, and are hypothesized as the underpinning mechanisms modulating the connection between neurotransmitters’ physiological changes and mental health conditions. That said, few studies have explored the molecular relationship between neurotransmitters’ physiological changes in the retina and brain-related conditions or mental health illness.

In view of the potential link between neurotransmitters in the brain and the eyes, ERG has successfully detected retina-related changes for various mental health conditions, including depressive disorders, panic disorders, ADHD, autism spectrum disorder, eating disorders, and schizophrenia [22]. Also, ERG has been used to detect changes related to suicidal behaviors among individuals with the major depressive disorder [7].

#### Neurotransmitters correlated with suicide or suicidal behaviors

Almost all neurotransmitters in the retina correlate with suicide [23, 24]. For instance, a recent review on neurotransmitter systems related to suicide using post-mortem studies reported that monoamines, glutamate, GABA, and endocannabinoids, neurotransmitters, enzymes, or receptors are correlated with suicide [24], albeit monoamines have been relatively well studied [25]. In fact, one of the first described molecular changes in individuals who are suicidal was the correlation between low serotonin levels and 5-hydroxy indoleacetic acid (5-HIAA) in cerebrospinal fluid (CSF) with suicidal behaviors [23, 26–28]. Further research has also supported this link: including the finding that altered levels of serotonin and serotonin signalling are correlated with suicidal behaviours [29], and binding of the serotonin_1A_ BP_F_ in the raphe nuclei was correlated with the lethality of a suicide attempt. Specifically, a higher lethality suicide attempts was linked with higher levels of binding of the serotonin_1A_ BP_F_ in the raphe nuclei compared to lower lethality attempts [30].

#### Neurotrophic factors correlated with suicide or suicidal behaviours

In addition to the neurotransmitters, there are neurotrophic factors that may influence neuronal connections in the retina, which have been correlated with suicide. One example is the differential expression patterns of the BDNF receptor TRKB between individuals who die by suicide and controls [29]. With neurotransmitters and neurotrophic factors in the retina correlating with suicide or suicidal behaviors, ERG is a potential non-invasive biomarker for suicidal behavior, although more studies are needed to establish this concept.

### A review of existing studies on ERG

A few published reports highlighted findings about the use of ERG to study suicidal behaviors, but no review has been conducted to synthesize these findings [7, 31–45]. Here, the available literature has proven very useful for new research by showing the use of ERG waveform changes in detecting suicidal behavior. It is with this background that we design this study to describe eligible studies on this theme and comment on the opportunities they provide as well as their limitations. Overall, we focused this review to summarize all the consistent results that have been described while acknowledging that studies with contradictory results reflect the complexity of this area of research.

### Methods

The present review included existing reports on suicide, suicidal behaviors and ERG. The present review followed the PRISMA guidelines [46]. All article types were included, and there was no restriction to the language in which the studies were published. The following databases were used: Ovid databases (i.e., Embase, APA PsycInfo, Ovid Emcare, Ovid Medline, and Epub Ahead of Print), PubMed, Web of Science, and CINAHL. The following keywords were used with assistance from the institutional librarian (Kaitryn Campbell) to develop a search strategy: (i) electroretinography (Electroretinography/ OR (electroretinography* OR electro-retinograph*).tw,kw,kw,id.) and (ii) suicidal behaviors (exp Suicide/ OR suicid*.tw,kw,kw,id.).

**Fig. 3:**
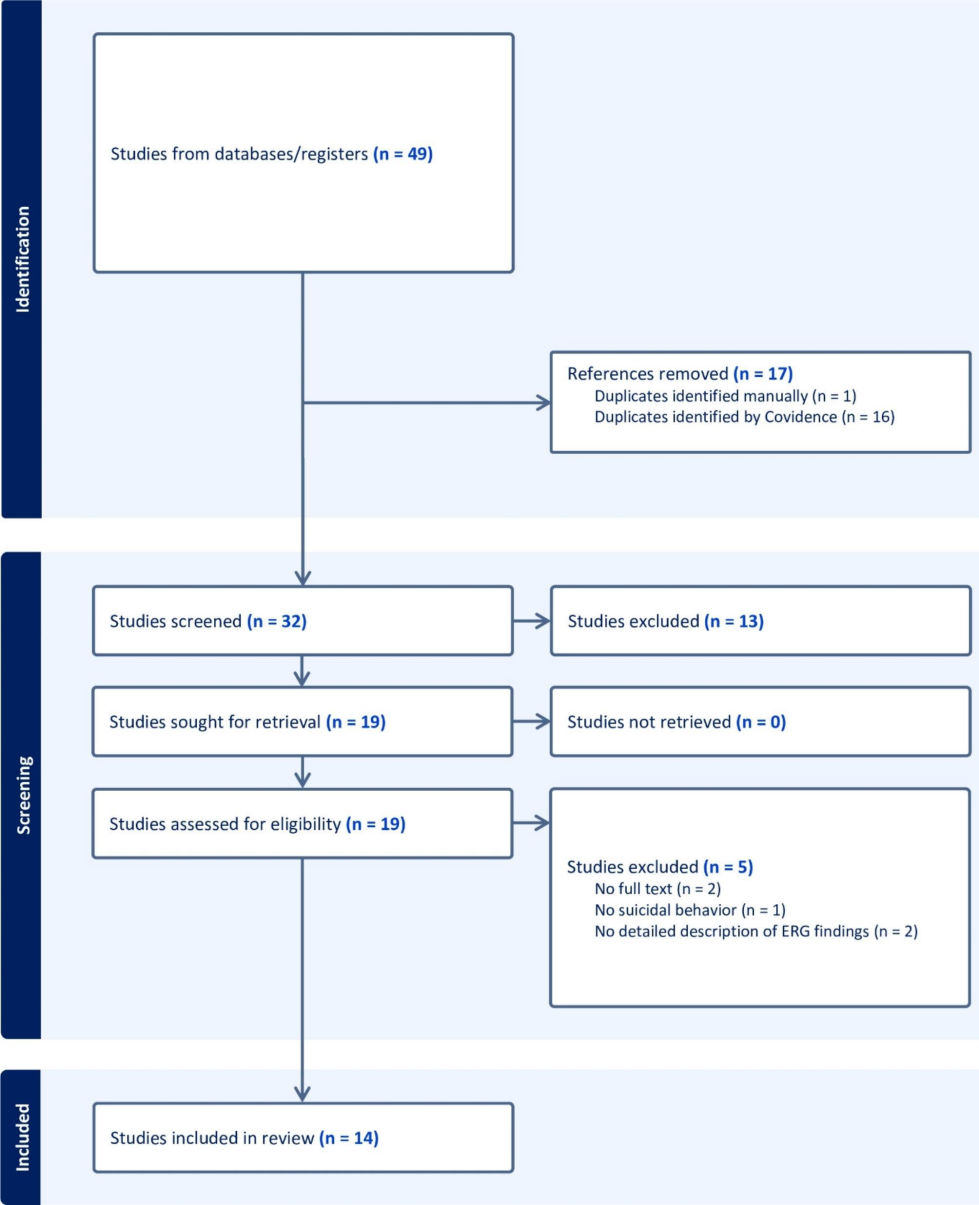
PRISMA flow diagram

Following the removal of duplicates, all identified articles were screened independently by pairs of researchers via Covidence. Screening was based on the following process (i) title and abstract, (ii) full text, and (iii) data extraction and cleaning (See Fig. 3). The following information was extracted by a pair of authors: Author, title, year of publication, study design, study group, countries where the study was conducted, sample size, medication before ERG, suicidal behaviors assessed, a method for assessing suicidal behaviors, ERG testing done, ERG findings, conclusion about ERG findings.

## Results

### Study selection

A total of 50 studies were identified from the different databases; 17 were duplicates. The remaining 33 articles’ titles and abstracts were screened, and 19 papers were considered eligible. After a full-text review, 14 articles were included in the current study. Two full texts could not be retrieved [31, 32]. JBI critical appraisal tools were used to assess the quality of the included study (Supplementary File 1). Overall, studies did a good job of describing patient conditions upon arrival and of describing the ERG findings. Many studies [29, 32–37] did not include an intervention or treatment, as assessing treatment was not the intent of these studies. However, this exclusion does not impact the results of the following findings as this review was only interested in ERG measures following suicide.

### Characteristics of the included studies

Among the included studies, 12 were case reports [34–45], one case series [33], and one cross-sectional study [7]. The studies were published between 1980 and 2022. (See Table 2).

**Table 2:** Findings from the different studies

Study ID	SD	Population description	Substance use before ERG (quantity if known)	ERG testing performed	ERG performance details	ERG findings	Comments about ERG finding
Zahn 1981 [41]	CR	A 25-year-old woman who attempted suicide by ingesting quinine vs. a normal control	Quinine (3.8–4.8 g)	Unspecified ERG type	ERG findings recorded on days 1, 2, 3, 6, 9, 10, 12, and at six months	- Day 1: amplitude of the a-wave was greater than that of the control, while the amplitude of the b-wave was less than the control. The implicit time for both the a and b waves was increased. The b-a amplitude ratio decreased with time but was normal only on days 3 to 6. - Days 3 and 6: a-wave amplitude was normal while b-waves were enlarged (more for the left than the right eye). - 6 months: b-wave amplitude decreased substantially by this date. - The a-wave implicit time reached normal by day 2, while the b-wave implicit time increased up to day 12. - OPs, compared to controls, was completely extinguished on day 9, improved on days 10 and 12 (though still decreased compared to the control) and remained diminished by six months.	- Had a limitation: difficult to accurately identify b-wave peaks - Depressed ERG was due to damaged bipolar or photoreceptor cells through secondary retinal ischemia
Mindel et al. 1981 [35]	CR	A 20-year-old woman attempted who died by suicide following ingesting of Vacor (a rat poison).	Vacor and Ergotamine (used to treat Vacor-induced orthostatic hypotension)	Unspecified ERG type	ERG performed 5 and 10 weeks after discontinuing ergotamine	- ERG waves were extinguished in both eyes	- The findings were inconclusive on the true cause of extinguished ERG waves, whether from Vacor, Ergotamine, or a combination of the two toxicities
Verdon 2008 [42]	CR	43-year-old woman evaluated nine months after attempting suicide by ingesting quinine	Quinine	Multifocal ERG (mERG) Full-field (Ganzfeld) ERG (ffERG)	ERG’s performed 9 months after the suicide attempt.	- Subnormal Rod’s responses to dim flash stimuli. - At both the scotopic and photopic SF - The a-wave amplitude exceeded the b-wave amplitude as the b-wave had selective reductions in both conditions (electronegative waveforms). - For the scotopic SF – normal a-wave amplitudes, but reduced b-wave amplitudes. - For the scotopic SF – normal a-wave implicit times and faster than normal b-wave implicit times - Phototopic SF- abnormal b-wave with a delayed implicit time. - Scoptopic OPs - abnormal with a single positive peak (corresponding in time to the positive peak in original waveform)	-
Treichel et al. 2004 [40]	CR	A 44-year-old woman attempted suicide in her car by ingesting methanol An age- and sex-matched control	Methanol	Unspecified ERG type	ERG measurements were performed 8, 18, and 26 h after admission, where patient was missing for 48 h prior to admission. ERG performed while individual was comatose.	- Postadmission intervals, all ERGs were similar. With a significant diminution of the b-wave, but the a-wave remained stable (apparently normal amplitudes and implicit times).	- Findings were similar to animal models following methanol ingestion
Brinton et al. 1980 [39]	CR	A 25-year-old woman who attempted suicide by ingesting quinine Vs. an age- and sex- matched control	Quinine (3.7–4.7 g)	Flashlight functional ERG	Light perception was lost 14.5 h after ingestion; first ERG was completed 18 h later. Other ERG tests performed day 2, day 12, and 6 months ERG obtained after 15 min of dark adaptation	- Slowing a-wave that returned to normal after two days. - a-wave depth increased and returned to normal after several days. - Loss of OPs with no tendency for recovery - b-wave decreased in size and returned toward normal after a first few days, but a late progressively decreased in size	- Quinine has direct toxicity to retinal cells. It is known to affect ganglion cells, pigment epithelium, and other retina layers. - The delayed a-wave (prolonged implicit time) when there was no light perception. Indicates evidence of an acute effect on photoreceptors cell layers. - The later decline in b-wave shows inner layer damage. This may be through vascular constriction or ischemia due to quinine.
Canning and Hague 1988 [44]	CR	28-year-old woman attempted suicide by ingesting quinine, presented 12 h after ingestion	Quinine (9 g)	Flash ERG (fERG) Pattern ERG (PERG)	First ERG was completed on day 1, then repeated several times over the course of 66 days	- Day 1: Normal amplitude to blue, red, and white stimuli, but reduced OPs. The pattern ERG was reduced - Over time, the amplitude reduced, but abnormal flicker response and pattern ERG persisted.	- Quinine must affect the initial electrically silent part of the retina that don’t contribute to the fERG waveforms e.g., the ganglion cells. This is due to having a normal early ERG in the presence of severe visual dysfunction. - The inner retina was mainly affected because the abnormal pattern ERG and reduced OPs during the same period in the patient. - “The pattern ERG was markedly reduced, and no pattern visual evoked response (VER) could be recorded.”
Simmons and Good 1998 [33]	CS	Two out of three patients presenting with CO poisoning attempted suicide. Case 1: a 43-year-old woman who attempted suicide and presented to hospital after three weeks Case 2: 59-year-old man who attempted suicide and presented ten years following a suicide attempt.	None	Flash ERGs (fERG) Pattern ERGs (PERG).		- Case 1: ERGs were normal from both eyes, the N95 component increased within the normal range. The P50 / N95 ratios were normal. - Case 2: The P50/ N95 ratio was significantly elevated and the fERGs were normal in both eyes.	- Normal fERG findings.
Traill et al. 2007 [36]	CR	A 29-year-old woman attempted suicide by ingesting quinine	Quinine (6 g)	fERG	ERG completed 13 years after the suicide attempt.	- Flat photopic response and a scotopic response with an absent b-wave	
Meshi et al. 2015 [38]	CR	A 59-year-old woman with a diagnosis of borderline personality disorder, benzodiazepine and alcohol use disorder attempted suicide by ingesting quinine and alcohol and arrived at the hospital three days after attempting suicide.	Quinine (12 g), benzodiazepine, alcohol. High dose beta-carotene was given 10 days post ingestion (and taken daily for 3 months), after the first ERG (day 6) but before ERGS completed on day 6, 39, and 142.	Full-field ERG (ffERG)	ERG were obtained 6-, 39, and 142-days post-ingestion.	- The amplitude and pattern for the scotopic and photopic responses in both eyes were diminished. - A significant reduction in both a-wave and b-wave indicating a primary insult to the photoreceptors. - During treatment, the ERG responses showed mild improvement in the a-wave amplitude in both eyes in the maximal scotopic conditions. Following treatment discontinuation, the a-wave of both eyes decreased. - The b-waves had no similar pattern and continued to deteriorate during follow-up. - Only the left eye single flash photopic responses demonstrated some improvement during treatment but decreased after treatment ceased.	- ERG showed transient increase in a-wave amplitude. A-wave decreased after treatment. o This improvement may be attributed to a positive effect of 9-cis beta-carotene treatment on the photoreceptors and the retinoid cycle - A limited, slow recovery of the ERG responses after they were almost completely absent over a 2-year follow-up. Thus, a short-term increase in the a-wave amplitudes may be the direct effect of treatment. - These changes may represent the natural course of ERG responses in quinine toxicity. - The lack of consistent increase in ERG b-wave responses during treatment o This may be because 9-cis beta-carotene impacts photoreceptors, which are not responsible for b-waves
Kohli 2021 [45]	CR	A 45-year-old man who attempted suicide by hanging and presented to the hospital after six months		Ganzfield ERG Patient was initially dark-adapted (DA) for 30 min and then Light Adapted (LA) ERGs were performed	ERG completed six months after suicide attempt	- Recording of the right eye done at DA 0.01 cd s m-2 showed a reduced amplitude a-wave; reduced b/a wave amplitude ratio at DA 3.0 and DA 10.0 cd s m-2. - The OPs on the DA 3.0 wave showed reduced amplitudes. - Light-adapted ERG also showed a reduced b/a wave amplitude ratio along with a reduced amplitude 30-Hz flicker.	- The dysfunction was post-phototransduction (at the level of the inner bipolar cells of the retina) due to having an electronegative ERG in the presence of a normal electrooculography (EOG). - Reduced OP waves suggest amacrine cells dysfunction - The cause of the inner retina cell dysfunction could be due to hypoperfusion.
Chen 2022 [34]	CR	A female, aged 19-year with a history of depression had a suicide attempt byg ingesting lorazepam and magnesium valproate	Lorazepam (19 mg) and Magnesium valproate (VPA) (60 g)	Multifocal ERG (mfERG)		- Based on the mfERG, P1 amplitude in the right was lower than in the left eye (more damage to right eye)	- Findings were inconclusive with ERG, and mingling was thought - Unable to ascertain which drug (lorazepam, VPA, or a combination of the two) led to these ERG findings
Miura 2022 [43]	CR	A 23-year-old man presented 3 months after attempting suicide by ingesting sildenafil	Sildenafil (2 g)	Full field photopic ERG (ffERG) Multifocal ERG (mfERG)	ERGS obtained 3 and 6 months after overdose DA 0.01, DA 3.0, LA 3.0, and LA 30 Hz flicker were performed	A slightly reduced amplitudes of full-field photopic ERGs; But normal amplitudes of the scotopic, maximum combined, and flicker ERGs. - Reduced multifocal ERGs (mfERGs), especially in the bilateral paracentral areas. - Complete normalization of ERG did not occur six months following treatment	- PDE5 inhibition in bipolar and ganglion cells and PDE6 inhibition at the photoreceptors may be associated with Sildenafil-induced visual impairment.
Bacon et al. 1988 [37]	CR	A 47-year-old man who presented 24 h after attempting suicide using quinine sulfate and flurazepam.	Quinine sulfate (10 g) and Flurazepam (600 mg)	Unspecified ERG type	Dark-adapted ERG was done 4 and 10 weeks after the overdose.	- Week 4: The left eye dark-adapted ERG was subnormal with the absence of the b wave. This suggested a proportionately greater loss of postsynaptic electrical activity than photoreceptor activity. - Week 10: b-wave amplitude had a small improvement.	- Within three days, the ERG approached normal values, but by the ninth day, the b wave had started to decrease and, six months later, remained essentially unchanged. Similar findings have been found in rabbit experiments.
Fountoulakis et al. 2004 [7]	Cx	A total of 52 patients (15 males, 35 females, mean age of 41.0 ± 11.4) with major depressive disorder (MDD)were enrolled in the study. The participants were divided into three groups, (1) No thoughts of death (n = 17), (2) nonspecific thoughts of death (n -= 23), and (3) suicidal ideations (n = 10) Among these, five participants had attempted suicide	Individuals free from medication for at least two weeks before initial assessment All 5 suicide attempts were performed by swallowing pills, drugs, or poison, but the type and quantity are unknown.	Flash ERG (fERG)		- No significant difference between the groups. - a-wave latency was lowest among individuals with suicidal ideation. - b-wave latency was highest among individuals with suicidal ideations	

SD = study design, CR = case report, CS = case series, Cx = Cross-sectional study

### Characteristics of the study participants

The age of the participants in the case reports and case series ranged between 19 and 59 years. However, the majority of the studied cases included individuals who were below 50 years, a finding that resonated with the cross-sectional study (mean age 41.0 ± 11.4). Most of the studies had female individuals who attempted suicide, and four had male individuals [33, 37, 43, 45]. Three studies reported a history of mental illness among the participants, and they included bipolar affective disorder [34], major depressive disorder [7, 34], and borderline personality disorder [38]. A total of 19 participants included in this review had attempted suicide.

### Medications used to attempt suicide

Six studies reported the use of quinine (above 3.7 g) as medication to attempt suicide [36, 38, 39, 41, 42, 44], one study each reported the use of methanol [40], ergotamine [35], carbon monoxide poisoning [33], sodium valproate [34], lorazepam [34], and flurazepam [37].

### Periods ERG was performed

A total of six studies followed up patients from the first day of the suicide attempt [37–39, 41, 43, 44], while five studies performed ERG five months after the suicidal attempt [33, 35, 36, 42, 45].

### ERG wave findings

The findings ranged from having completely extinguished waves to fluctuating waves following a suicidal attempt. The following waves were affected as shown in Table 3.

**Table 3:** Wave forms for individuals with suicidal behaviors

Wave	Measure	Responses
a-wave	Amplitude	Normal [37, 40, 42]
		Increased in the first two days that later became normal [39, 41]
		Reduced [38, 45]
	Implicit time	Normal [40, 42]
	Latency	Decreased [7]
b-wave	Amplitude	Reduced [37–42], tried to increase after initial decrease but eventually remained decreased.
	Implicit time	Increased or faster than normal [41, 42]
		Absent [36]
	Latency	Increased [7]
b-a amplitude ratio		Decreased with time [41, 45]
Oscillatory potential		Decreased [39, 41, 44, 45] and were completely extinguished after [39, 41]
Overall ERG patterns		Reduced [43, 44]
		Normal [33]

Note: Some studies refer to latency and implicit time as the same thing, defining them both as the time it takes to reach the trough of the a-wave and peak of the b-wave [47]. While one study measured latency [7], it is unclear whether they were measuring the time from stimulus to onset of response or the time from stimulus to the trough and peak of the waves.

Based on the included papers, most studies reported normal amplitude and implicit time of the a-waves. Still, the latency was lower compared to individuals without suicidal behaviors. The b-waves amplitude was reduced, but the implicit time and latency were increased. The b-a amplitude ratio and oscillatory potential were decreased.

## Discussion

The finding from the various included studies indicates a correlation between ERG waveform changes and suicidal behaviors. However, most of the included studies were case reports and a case series. More robust studies with advance methods are needed to better understand the electrophysiological changes associated with suicidal behaviors and their clinical translation. Future studies should address the following methodological issues, taking into consideration the observations highlighted.

- The findings in the included studies might have been cofounded by hypoxia-related neuronal defects in the retina, which could influence changes in neurotransmission and the waves displayed by the ERG. It is necessary to address this issue by controlling for this effect or excluding individuals with possible hypoxia-related suicide attempts due to hanging, use of methanol, rat poison (such as Vacor), quinine, sildenafil, among others. Also, because the OP waves depend on some vascular response in the retina, the wave’s shape is affected by vascular diseases such as diabetic retinopathy on ERG [41]. Therefore, we recommend screening individuals for diabetes and potential vascular diseases that may affect the retina.
- Individuals who attempt using sildenafil may also be excluded due to PDE5 inhibition in bipolar and ganglion cells and PDE6 inhibition at the photoreceptors that affect the ERG wave forms [43].
- Individuals who have attempted suicide with quinine-related compounds should also be excluded because the vast available literature among animals and humans’ studies showed its effect on the ERG findings [38, 44]. However, this should be done in consideration that the studies in humans were case reports, and no controlled study was assessed. In addition, not all the different parameters of the ERG were explored and affected. For instance, the b/a wave ratio and OP among others were not explored, despite evidence suggesting that they can detect changes in neurotransmitters in the retina, which could potentially be correlated with suicidal behaviors.
- The use of 9-cis-beta-Carotene in managing quinine-related ERG changes [38] may indicate that the vitamin can be used in suicidal behavior management. Other studies have suggested that 9-cis-beta-Carotene is significantly helpful in managing suicidal behaviors and depression [48–50]. The effect is related to the antioxidant properties of the vitamin [49]. The study did not check the impacts of the vitamin on OP and other important ERG parameters to make a satisfactory conclusion on its importance on the neurotransmitters or waves in the retina [38].
- Individuals who use carbon monoxide poisoning should also be considered excluded because of the potential effects it had on the ERG findings [33]. However, the findings were reversed and normal after three weeks following the suicidal attempt.

### Study limitations

The findings of this review should be interpreted with caution for the following reasons. (i) majority of the studies are case reports and lack sufficient statistical power to confirm the actual effect or changes in the ERG waves caused by suicidal behaviors. It also makes it hard to distinguish the effects of cofounders to clarify whether the effect on ERG wave is due to medications, suicidal behaviors, or psychiatric illness.(ii) There was no actual link between neurotransmitters in the retina and specific regions responsible for suicidal behaviors in the brain. This makes the conclusions on the findings about ERG correlates of suicidal behaviors difficult since no study has explored such results to implicate specific brain regions We recommend that future studies explore this link. (iii) The OP waves are not specific to retina neurotransmitters and may not be specific to inhibitory or excitatory cells in the amacrine cells. The amacrine cells have many neurotransmitters that are correlated with suicidal behaviors. The lack of specificity of the waves to distinguish between the neurotransmitters makes detecting suicidal behaviors difficult. We recommend that future researchers determine the effect of individual neurotransmitters on the OP waves and other waves of the ERG. This will advance the science of eliciting potential biomarker that is more specific and reliable. (iv) Only few studies have explored ERG use among individuals with suicidal behaviors. These studies are limited because of cross-sectional design and use of small sample, and causality can not be inferred. We recommend future prospective studies s with larger sample sizes to enable the detection of the small changes in the different wave measurements, such as latency, and amplitude, among others, (v) Distinguishing between the severity of suicide from depression is complex using ERG because of the high level of similarities between the neurotransmitters involved in the two conditions. Future researchers show study suicidal behaviors among individuals with and without depression to detect the ERG differences in the two conditions using a control. (vi) Many studies were performed months after the suicidal attempt, and it is hard to determine the effect or correlates of suicidal behavior on the ERG. A follow-up study proximal to the suicidal attempt may yield better results for at least six months post attempt since ERG changes related to suicide were observed at six months. (vii) Most studies involved females, and significant differences between genders may need to be explored. Also, the extreme of ages were not studied. Therefore, future studies should consider matching the populations studied and controlling for variables that may confound, such as gender, age, and medication use, among others. (viii) The ISCEV standards for ERG use are updated regularly, making ERG comparisons difficult as the case studies used for this review span decades, including before the first ISCEV standards were published.

## Conclusion

The majority of the studies reported normal amplitude and implicit time of the a-waves, but the latency was lower compared to individuals without suicidal behaviors. The b-waves amplitude was reduced, but the implicit time and latency were increased. The b-a amplitude ratio and oscillatory potential were decreased. This may indicate the effects of suicidal behaviors on the inner nucleus layers of the retina with a potential reduction of the depolarization of the inner retina Muller, bipolar cells, and amacrine cells. An effect may be related to some of the neurotransmitters within the layers. Among these neurotransmitters, many are involved in suicidal behaviors. While some promising results have been highlighted, more research work is needed to translate the evidence from existing literature into clinical practice and to advance the potential utility of ERG findings as biomarker in suicidology. Notably, further studies are needed to explore the relationship between ERG waves, retina neurotransmitters, and suicidal behaviors.

### Electronic supplementary material

Below is the link to the electronic supplementary material.

Supplementary Material 1

## Data Availability

The data used in the present manuscript is available on request from the corresponding author.
