# Supplementary material for: Electroretinography and suicidal behaviors: a systematic review
Source: BMC Psychiatry. 2023 Dec 15;23:948. doi: 10.1186/s12888-023-05453-w (PMC10724949; doi:10.1186/s12888-023-05453-w)
Supplement: Supplementary file 1 — Supplementary Material 1 [file 12888_2023_5453_MOESM1_ESM.docx]

**Supplementary File 1: JBI appraisal tools for the included studies**

| **JBI Critical Appraisal for Case Reports[1]** | | | | | | | | |
| --- | --- | --- | --- | --- | --- | --- | --- | --- |
| **Study ID** | Were patient’s demographic characteristics clearly described? | Was the patient’s history clearly described and presented as a timeline? | Was the current clinical condition of the patient on presentation clearly described? | Were diagnostic tests or assessment methods and the results clearly described? | Was the intervention(s) or treatment procedure(s) clearly described? | Was the post-intervention clinical condition clearly described? | Were adverse events (harms) or unanticipated events identified and described? | Does the case report provide takeaway lessons? |
| Chen 2022 [2] | Yes | Yes | Yes | Yes | Yes | Yes | N/A | No |
| Mindel et al. 1981[3] | Yes | Yes | Yes | No; ERG results described but other tests not detailed | Yes | Yes | Yes | Yes |
| Traill et al 2007 [4] | Yes | Yes | Yes | No | N/A | Yes | Yes | Yes |
| Bacon et al. 1988 [5] | Yes | No | Yes | Yes | N/A; no treatment given | Yes; described change over time | Yes | Yes |
| Meshi et al. 2015 [6] | Yes | Yes | Yes | Yes | Yes | Yes | Yes | Yes |
| Brinton et al. 1980 [7] | Yes | Yes | Yes | Yes | Yes | Yes | Yes | Yes |
| Treichel et al. 2004 [8] | Yes | Yes | Yes | Yes | N/A  (patient deceased shortly after arrival; no treatment) | N/A | N/A | Yes |
| Zahn 1981[9] | Yes | No; history merely stated as “normal” | Yes | Yes | N/A; no treatment given | Yes; described change over time | N/A | Yes |
| Verdon 2008 [10] | Yes | No; history details present but limited | Yes | Yes | N/A; no treatment given | N/A | N/A | No |
| Miura 2022 [11] | Yes | Yes | Yes | Yes | N/A; no treatment given | N/A | N/A | No |
| Canning and Hague 1988 [12] | Yes | Yes | Yes | Yes | N/A; no treatment given | N/A | N/A | No |
| Kohli 2021[13] | Yes | Yes | Yes | Yes | N/A; no treatment given | N/A | N/A | No |

| **JBI Critical Appraisal for Case Series[14]** | | | | | | | | | | |
| --- | --- | --- | --- | --- | --- | --- | --- | --- | --- | --- |
| **Study ID** | Were there clear criteria for inclusion in the case  series? | Was the condition measured in a standard, reliable way for all participants included in the case series? | Were valid methods used for identification of the condition for all participants included in the case series? | Did the case series have consecutive inclusion of participants? | Did the case series have complete inclusion of participants? | Was there clear reporting of the demographics of the participants in the study? | Was there clear reporting of clinical information of the participants? | Were the outcomes or follow up results of cases clearly reported? | Was there clear reporting of the presenting site(s) /clinic(s) demographic information? | Was statistical analysis appropriate? |
| Simmons and Good 1998 [26] | No | Unclear | No | No | Unclear | Yes | Yes | Yes | Yes | N/A |

| **JBI Critical Appraisal for Analytical Cross-Sectional Studies[1]** | | | | | | | | |
| --- | --- | --- | --- | --- | --- | --- | --- | --- |
| **Study ID** | Were the criteria for inclusion in the sample clearly defined? | Were the study subjects and the setting described in detail? | Was the exposure measured in a valid and reliable way? | Were objective, standard criteria used for measurement of the condition? | Were confounding factors identified? | Were strategies to deal with confounding factors stated? | Were the outcomes measured in a valid and reliable way? | Was appropriate statistical analysis used? |
| Fountoulakis et al 2004 [15] | Yes | Yes | Yes | Yes | Yes | Yes | Yes | Yes |

**References**

1. Moola S, Munn Z, Tufanaru C, Aromataris E, Sears K, Sfetcu R, Currie M, Qureshi R, Mattis P, Lisy K: **Chapter 7: Systematic reviews of etiology and risk**. *Joanna briggs institute reviewer’s manual The Joanna Briggs Institute* 2017, **5**:217-269.

2. Chen X, Di H, Hong Y, Zhang C: **Late-stage sequela of an isolated optic neuropathy after acute magnesium valproate overdose: A case report**. *Eur J Ophthalmol* 2020:1120672120974943.

3. Mindel JS, Rubenstein AE, Franklin B: **Ocular ergotamine tartrate toxicity during treatment of Vacor-induced orthostatic hypotension**. *Am J Ophthalmol* 1981, **92**(4):492-496.

4. Traill A, Patmaraj R, Zamir E: **Quinine iris toxicity**. *Arch Ophthalmol* 2007, **125**(3):430.

5. Bacon P, Spalton DJ, Smith SE: **Blindness from quinine toxicity**. *Br J Ophthalmol* 1988, **72**(3):219-224.

6. Meshi A, Belkin A, Koval T, Kornhouser T, Assia EI, Rotenstreich Y: **An experimental treatment of ocular quinine toxicity with high-dose 9-cis Beta-carotene**. *Retin Cases Brief Rep* 2015, **9**(2):157-161.

7. Brinton GS, Norton EW, Zahn JR, Knighton RW: **Ocular quinine toxicity**. *Am J Ophthalmol* 1980, **90**(3):403-410.

8. Treichel JL, Murray TG, Lewandowski MF, Stueven HA, Eells JT, Burke JM: **Retinal toxicity in methanol poisoning**. *Retina* 2004, **24**(2):309-312.

9. Zahn JR, Brinton GF, Norton E: **Ocular quinine toxicity followed by electroretinogram, electro-oculogram, and pattern visually evoked potential**. *American journal of optometry and physiological optics* 1981, **58**(6):492-498.

10. Verdon W: **Clinical electrophysiology in quinine induced retinal toxicity**. *Optom Vis Sci* 2008, **85**(1):17-26.

11. Miura G, Baba T, Hashimoto R, Yamamoto S: **Long-term follow-up of retinal morphology and physiology after 2000 mg sildenafil overdose as a means of attempted suicide: a case report**. *BMC Ophthalmol* 2022, **22**(1):216.

12. Canning CR, Hague S: **Ocular quinine toxicity**. *Br J Ophthalmol* 1988, **72**(1):23-26.

13. Kohli P, Jayasri KN, Rupa A, Kumar M, Kowsalya A: **Electrophysiological and neuroimaging findings in a patient who developed visual loss after attempted suicide by hanging**. *Doc Ophthalmol* 2021, **143**(3):331-337.

14. Munn Z, Barker TH, Moola S, Tufanaru C, Stern C, McArthur A, Stephenson M, Aromataris E: **Methodological quality of case series studies: an introduction to the JBI critical appraisal tool**. *JBI Evid Synth* 2020, **18**(10):2127-2133.

15. Fountoulakis KN, Iacovides A, Fotiou F, Nimatoudis J, Bascialla F, Ioannidou C, Kaprinis G, Bech P: **Neurobiological and psychological correlates of suicidal attempts and thoughts of death in patients with major depression**. *Neuropsychobiology* 2004, **49**(1):42-52.
